# Supplementary material for: CryoEM structures reveal how the bacterial flagellum rotates and switches direction
Source: Nat Microbiol. 2024 Apr 17;9(5):1271–81. doi: 10.1038/s41564-024-01674-1 (PMC11087270; doi:10.1038/s41564-024-01674-1)
Supplement: Supplementary file 1 — Supplementary Tables 1 and 2. [file 41564_2024_1674_MOESM1_ESM.pdf]

nature microbiology

Article https://doi.org/10.1038/s41564-024-01674-1

CryoEM structures reveal how the bacterial flagellum rotates and switches direction

In the format provided by the authors and unedited

Supplementary Tables

Supplementary Table 1

	CCW C-ring (EMDB: EMD-41100) (PDB:8T8O)	MS-ring (EMDB: EMD-41101) (PDB:8T8P)	CW C-ring (EMD-43256) (PDB:8VIB)	CW C-ring with extra density (EMD-43258) (PDB:8VID)
Data Collection				
Microscope	Thermo Fisher FEI Titan G4	Thermo Fisher FEI Titan G4	Thermo Fisher FEI Titan G4	Thermo Fisher FEI Titan G4
Detector	Gatan K3	Gatan K3	Gatan K3	Gatan K3
Magnification	105,000x	105,000x	105,000x	105,000x
Voltage (kV)	300	300	300	300
Electron exposure ($e^-/Å^2$)	51.557	51.557	56.323	59.242
Defocus range (μm)	-1.0 to -2.0	-1.0 to -2.0	-1.0 to -2.1	-0.9 to -2.3
Pixel size (Å)	0.818	0.818	0.818	0.818
Total micrographs taken	34,381	34,381	35,552	26,130
Data Processing				
Micrographs used for processing	34,275	34,275	35,552	26,130
Symmetry imposed	C34	C11	C34	C34
Initial particle images	295,031	295,031	43,741	59,404
Final particle images	51,268	16,411	7,201	11,106
Map resolution (Å)	3.98	3.42	4.6	5.9
FSC threshold	0.143	0.143	0.143	0.143
Model Refinement				
Initial model used (PDB)	Ab-initio	PDB: 8FTF[10]	PDB: 8T8O	PDB: 8T8O
Model resolution (Å)	3.9 (masked), 3.9 (unmasked)	3.4 (masked), 3.5 (unmasked)	4.6 (masked), 10 (unmasked)	5.9 (masked), 10 (unmasked)
Model composition				
Total atoms	205,020	69,025	151,198	151,198
Protein residues	30,396	9,009	30,362	30,362
Validation				
RMSD Bond lengths (Å)	0.007	0.009	n/a	n/a
RMSD Bond angles (°)	0.984	0.916	n/a	n/a
Rotamer outliers (%)	1.04	0.21	n/a	n/a
Ramachandran plot			n/a	n/a
Favored (%)	95.06	96.53	n/a	n/a
Allowed (%)	4.94	2.86	n/a	n/a
Disallowed (%)	0	0.61	n/a	n/a

Supplementary Table 1. Cryo-EM data collection, processing, model refinement, and validation statistics.

Supplementary Data Table 2

Subunit	Residues	Domain Name	Name(s) in literature	Location	Refs
FliF	$FliF_{1-15}$	$FliF_N$	FliF N-terminus	cytoplasm	
	$FliF_{15-50}$	TM1	TM1	M-ring	
	$FliF_{50-106}$	RBM1	RBM1	S-ring	[5]
	$FliF_{107-124}$	none (linker)	none	S-ring	
	$FliF_{125-228}$	RBM2	RBM2	S-ring	[5]
	$FliF_{228-273}$	RBM3a	RBM3a	S-ring	[5]
	$FliF_{273-379}$	β-collar	β-collar	S-ring	[5]
	$FliF_{379-437}$	RBM3b	RBM3b	S-ring	[5]
	$FliF_{438-501}$	TM2	TM2	M-ring	
	$FliF_{502-513}$	none (linker)	none	cytoplasm	
	$FliF_{514-560}$	$FliF_C$	$FliF_C$	C-ring	[34,36]
FliG	$FliG_{1-67}$	$FliG_{D1}$	$FliG_N$	C-ring	[34,36]
	$FliG_{68-72}$	$FliG_{L1}$	$Helix_{NM}$	C-ring	[38]
	$FliG_{73-99}$	$FliG_{D2}$	$Helix_{NM}$, H_6 & H_7, $G_N\alpha4$	C-ring	[34,36,38]
	$FliG_{100-106}$	$FliG_{L2}$	$loop_M$	C-ring	[38]
	$FliG_{107-186}$	$FliG_{D3}$	$FliG_M$, ARM_M, $FliG_M$, domain I	C-ring	[30,38]
	$FliG_{187-195}$	$FliG_{L3}$	$loop_C$, $helix_{MC}$	C-ring	[35,38]
	$FliG_{196-233}$	$FliG_{D4}$	ARM_C, $FliG_{CN}$	C-ring	[35,38]
	$FliG_{234-242}$	$FliG_{L4}$	none	C-ring	
	$FliG_{243-331}$	$FliG_{D5}$	$FliG_C$, $FliG_{CC}$, domain II	C-ring	[30,33,35]
FliM	$FliM_{1-16}$	$FliM_N$	$FliM_N$, $FliM_{16}$ (residues 1-16)	C-ring	[73]
	$FliM_{17-30}$	$FliM_N$	none	C-ring	
	$FliM_{31-50}$	$FliM_{L1}$	none	C-ring	
	$FliM_{51-230}$	$FliM_{mid}$	FliM, $FliM_{mid}$, $FliM_M$	C-ring	[32,74]
	$FliM_{231-256}$	$FliM_{L2}$	none	C-ring	
	$FliM_{257-330}$	$FliM_C$	$FliM_C$, SpoA domain	C-ring	[39]
FliN	$FliN_{1-44}$	$FliM_N$	none	C-ring	
	$FliN_{45/59/63-137}$	$FliN_C$	FliN, SpoA domain	C-ring	[39,42]

Supplementary Table 2. Domain nomenclature of FliF, FliG, FliM, and FliN. Residues that belong to structurally defined domains in the assembled C-ring are indicated. A subset of the historical name(s) for these regions of sequence are indicated. Select literature citations are included to illustrate settings where other names are used. As the mapping of domain names is meant to assist in comparison to past structural work, these citations focus on the nomenclature used in comparator structures for C-ring analysis rather than the first use of the domain name in the literature. RBM, ring-building motif; TM, transmembrane.
