# Supplementary material for: CryoEM structures reveal how the bacterial flagellum rotates and switches direction
Source: Nat Microbiol. 2024 Apr 17;9(5):1271–81. doi: 10.1038/s41564-024-01674-1 (PMC11087270; doi:10.1038/s41564-024-01674-1)
Supplement: Supplementary file 2 — Reporting Summary [file 41564_2024_1674_MOESM2_ESM.pdf]

nature portfolio

Corresponding author(s): T. M. Iverson

Last updated by author(s): Feb 27, 2024

Reporting Summary

Nature Portfolio wishes to improve the reproducibility of the work that we publish. This form provides structure for consistency and transparency in reporting. For further information on Nature Portfolio policies, see our Editorial Policies and the Editorial Policy Checklist.

Statistics

For all statistical analyses, confirm that the following items are present in the figure legend, table legend, main text, or Methods section.

n/a	Confirmed	
☐	☒	The exact sample size (*n*) for each experimental group/condition, given as a discrete number and unit of measurement
☒	☐	A statement on whether measurements were taken from distinct samples or whether the same sample was measured repeatedly
☒	☐	The statistical test(s) used AND whether they are one- or two-sided<br>*Only common tests should be described solely by name; describe more complex techniques in the Methods section.*
☒	☐	A description of all covariates tested
☒	☐	A description of any assumptions or corrections, such as tests of normality and adjustment for multiple comparisons
☒	☐	A full description of the statistical parameters including central tendency (e.g. means) or other basic estimates (e.g. regression coefficient) AND variation (e.g. standard deviation) or associated estimates of uncertainty (e.g. confidence intervals)
☒	☐	For null hypothesis testing, the test statistic (e.g. *F*, *t*, *r*) with confidence intervals, effect sizes, degrees of freedom and *P* value noted<br>*Give P values as exact values whenever suitable.*
☒	☐	For Bayesian analysis, information on the choice of priors and Markov chain Monte Carlo settings
☒	☐	For hierarchical and complex designs, identification of the appropriate level for tests and full reporting of outcomes
☒	☐	Estimates of effect sizes (e.g. Cohen's *d*, Pearson's *r*), indicating how they were calculated

Our web collection on statistics for biologists contains articles on many of the points above.

Software and code

Policy information about availability of computer code

Data collection	EPU version 3.0.0.4164 was used for the CCW dataset. EPU version 3.5.1.6034 was used for both CW datasets.
Data analysis	For map refinement CryoSPARC 4.2.1 was used,<br>For model building Phenix 1.20.1-4, Coot 0.9.8.8, MolProbity, UCSF Chimera 1.17.2, AlphaFold 2.3.2 was used. For illustrations and animations UCSF ChimeraX 1.7, Adobe llustrator 27.7, Blender 3.5, Molecular Node 2.8 was used.

For manuscripts utilizing custom algorithms or software that are central to the research but not yet described in published literature, software must be made available to editors and reviewers. We strongly encourage code deposition in a community repository (e.g. GitHub). See the Nature Portfolio guidelines for submitting code & software for further information.

Data

Policy information about availability of data

All manuscripts must include a data availability statement. This statement should provide the following information, where applicable:

- Accession codes, unique identifiers, or web links for publicly available datasets
- A description of any restrictions on data availability
- For clinical datasets or third party data, please ensure that the statement adheres to our policy

Source data (uncropped micrograph used for Figure 1b) are available with this publication. All raw, processed, and interpreted data that support the findings of this study are available in public repositories. Raw micrographs have been deposited with EMPIAR (https://www.ebi.ac.uk/empiar/) and accession codes EMPIAR-11597,

EMPIAR-11891, and EMPIAR-11892. CryoEM maps have been deposited at the EMDB (https://www.ebi.ac.uk/emdb/) with the accession codes EMD-41100, EMD-41101, EMD-41102, EMD-41103, EMD-41104, EMD-43256, EMD-43258, EMD-43327, and EMD-43328. Atomic coordinates of the 34-mer CCW C-ring and the 33-mer MS-ring have been deposited at the Protein Data Bank (www.rcsb.org) with the accession codes rcsb.org/structure/8t8o and rcsb.org/structure/8t8p. Atomic coordinates for the single subunit of the isolated CW-locked C-ring are deposited with the accession code rcsb.org/structure/8vib, the 34-mer isolated CW-locked C-ring are deposited with accession code rcsb.org/structure/8vid. Coordinates for a single subunit of the CW-locked C-ring bound to a partner protein have the accession code rcsb.org/structure/8vkq, and the 34-mer of the CW-locked C-ring bound to a partner protein have the accession code rcsb.org/structure/8vkr. Previously reported structures or computational models used to support this work are: Thermotoga maritima FliG (rcsb.org/structure/1lkv30; rcsb.org/structure/5tdy34), Helicobacter pylori FliG (rcsb.org/structure/3usw31, rcsb.org/structure/4fq032), T. maritima FliN (rcsb.org/structure/1yab42), S. enterica FliM:FliN fusion (rcsb.org/structure/4yxb39), S. enterica flagellar basal body (rcsb.org/structure/7cgo12), A. aeolicus MotA (rcsb.org/structure/8gqy71), C. jejunji MotA/B (rcsb.org/structure/6ykm65), C. sporogenes MotA/B (rcsb.org/structure/6ysf64), B. subtilis MotA/B (rcsb.org/structure/6ysl64), E. coli YcgR (rcsb.org/structure/5y6h56), T. maritima CheY-FliM1-16 (rcsb.org/structure/4iga57), E. coli quinol:fumarate reductase (rcsb.org/structure/1kf658), and FliO (alphafold.ebi.ac.uk/entry/A0A5C2LXN829).

Research involving human participants, their data, or biological material

Policy information about studies with human participants or human data. See also policy information about sex, gender (identity/presentation), and sexual orientation and race, ethnicity and racism.

Reporting on sex and gender	No human research participants were involved.
Reporting on race, ethnicity, or other socially relevant groupings	No human research participants were involved.
Population characteristics	No human research participants were involved.
Recruitment	No human research participants were involved.
Ethics oversight	No human research participants were involved.

Note that full information on the approval of the study protocol must also be provided in the manuscript.

Field-specific reporting

Please select the one below that is the best fit for your research. If you are not sure, read the appropriate sections before making your selection.

☒ Life sciences ☐ Behavioural & social sciences ☐ Ecological, evolutionary & environmental sciences

For a reference copy of the document with all sections, see nature.com/documents/nr-reporting-summary-flat.pdf

Life sciences study design

All studies must disclose on these points even when the disclosure is negative.

Sample size	For the wild-type FliFGMN in the CCW pose, 295,031 particles from 34,381 movies were used. For the CW-locked FliFGMN, 43,741 particles from 35,552 movies were used. For the CW-locked FliFGMN bound to a regulator, 59,404 particles from 26,130 movies were used.
Data exclusions	For all datasets, CTF fit above 10Å was excluded as they did not provide high resolution information. For wild-type FliFGMN in the CCW pose, in 2D classification all contaminant protein and low signal to noise ratio particles were removed, in heterogeneous refinement any remaining particles that was not a MSC-ring was removed by 3D alignment and in final refinement of the C-ring, MS-rings was removed and for the MS-ring, the C-ring particles were removed by particle subtraction.<br>For FliFGMN in the CW pose, all contaminant protein and low signal to noise ratio particles were removed in 2D classification and heterogeneous refinement, and the final refinement of the C-ring was performed by masking three subunits of the C-ring to obtain high resolution.<br>For FliFGMN in the CW pose with a regulator, all contaminant protein and low signal to noise ratio particles were removed in 2D classification and heterogeneous refinement, and the final refinement of the C-ring was performed by masking three subunits of the C-ring to obtain high resolution.
Replication	For the wild-type FliFGMN in the CCW pose, 295,031 protein complexes from one prepared grid was used and the calculations were performed two independent times. For the CW-locked FliFGMN, 43,741 protein complexes from one prepared grid was used and the calculations were performed once. For the CW-locked FliFGMN bound to a regulator, 59,404 protein complexes from one prepared grid was used and the calculations were performed once.
Randomization	Randomization was not performed.
Blinding	Blinding was not performed.

Reporting for specific materials, systems and methods

We require information from authors about some types of materials, experimental systems and methods used in many studies. Here, indicate whether each material, system or method listed is relevant to your study. If you are not sure if a list item applies to your research, read the appropriate section before selecting a response.

Materials & experimental systems

n/a	Involved in the study
☒	☐ Antibodies
☒	☐ Eukaryotic cell lines
☒	☐ Palaeontology and archaeology
☒	☐ Animals and other organisms
☒	☐ Clinical data
☒	☐ Dual use research of concern
☒	☐ Plants

Methods

n/a	Involved in the study
☒	☐ ChIP-seq
☒	☐ Flow cytometry
☒	☐ MRI-based neuroimaging

Plants

Seed stocks	N/A
Novel plant genotypes	N/A
Authentication	N/A
